# Comparative Mitochondrial Genomic Analysis Robustly Supported That Cat Tapeworm *Hydatigera taeniaeformis* (Platyhelminthes: Cestoda) Represents a Species Complex

**DOI:** 10.3389/fvets.2022.931137

**Published:** 2022-06-22

**Authors:** Hui-Mei Wang, Rong Li, Yuan-Ping Deng, Guo-Hua Liu, Yi-Tian Fu

**Affiliations:** Research Center for Parasites and Vectors, College of Veterinary Medicine, Hunan Agricultural University, Changsha, China

**Keywords:** cat tapeworm, *Hydatigera taeniaeformis*, species complex, mitochondrial genome, comparative analaysis

## Abstract

*Hydatigera taeniaeformis* is one of the most common intestinal tapeworms that has a worldwide distribution. In this study, we sequenced the complete mitochondrial (mt) genome of *H. taeniaeformis* from the leopard cat (designated HTLC) and compared it with those of *H. taeniaeformis* from the cat in China (designated HTCC) and Germany (designated HTCG). The complete mt genome sequence of HTLC is 13,814 bp in size, which is 167 bp longer than that of HTCC and is 74 bp longer than that of HTCG. Across the entire mt genome (except for the two non-coding regions), the sequence difference was 3.3% between HTLC and HTCC, 12.0% between HTLC and HTCG, and 12.1% between HTCC and HTCG. The difference across both nucleotide and amino acid sequences of the 12 protein-coding genes was 4.1 and 2.3% between the HTLC and HTCC, 13.3 and 10.0% between the HTLC and HTCG, and 13.8 and 10.6% between the HTCC and HTCG, respectively. Phylogenetic analysis based on concatenated amino acid sequences of 12 protein-coding genes showed the separation of *H. taeniaeformis* from different hosts and geographical regions into two distinct clades. Our analysis showed that the cat tapeworm *H. taeniaeformis* represents a species complex. The novel mt genomic datasets provide useful markers for further studies of the taxonomy and systematics of cat tapeworm *H. taeniaeformis*.

## Highlights

- The complete mitochondrial (mt) genome of *Hydatigera taeniaeformis* from the leopard cat in China is 13,814 bp in size.
- Phylogenetic analysis showed the separation of *H. taeniaeformis* from different hosts and geographical regions into two distinct clades.
- The molecular evidence presented here supports the hypothesis that *H. taeniaeformis* represents a species complex.

## Introduction

Tapeworms (Platyhelminthes: Cestoda) are one of the major groups of parasitic flatworms that are passively transmitted between hosts and parasitize virtually every vertebrate species (1). The adults of tapeworms live in the digestive tract of all groups of vertebrates, whereas larvae occur in different organs and body cavities of invertebrates and vertebrates (2). Tapeworms cause neglected diseases that can be fatal and are a major impediment to socioeconomic development (3, 4). Cestoda is a large animal class that is currently classified into 19 orders with ~5,000 valid species (5).

The family Taeniidae is a medically important group of tapeworms that generally consists of four valid genera, *Taenia, Echinococcus, Hydatigera*, and *Versteria*. Based on morphological features, some authors have recognized that the genus *Hydatigera* is valid, whereas others have treated this genus as a junior synonym of the genus *Taenia*. In a recent revision, the resurrection of the genus *Hydatigera* was proposed based on molecular evidence (6). The cat tapeworm *H. taeniaeformis* (formerly referred to as *T. taeniaeformis*), the type species of the genus *Hydatigera*, is the most common tapeworm of cats and rats (7). Although the zoonotic significance of this tapeworm remains unclear, it has also been recorded in humans in unusual circumstances (8). The correct identification and differentiation of cat tapeworm *H. taeniaeformis* have important implications for studying its epidemiology, systematics, and population genetics. Many studies have shown that several criteria (e.g., infectivity, development, morphology, and biochemistry) differ between *H. taeniaeformis* from different geographical origins (9–12). Based on morphological analysis, *H. taeniaeformis* was considered a complex of three cryptic species (13). Phylogenetic analyses of the *H. taeniaeformis* based on sequences of nuclear 18S rRNA and mitochondrial (mt) *cox*1 DNA also strongly support the presence of three distinct clades (13).

The mitochondrial genome (containing 36–37 genes) has been considered a useful marker for the species identification and differentiation of many tapeworms (14–16). The complete mt genomes of *H. taeniaeformis* from cats in China and Germany have been sequenced and supported that they represent different species (17), but it is yet unknown whether *H. taeniaeformis* from leopard cats represents third cryptic species. Therefore, in this study, we (i) sequenced the full mt genome sequence of *H. taeniaeformis* from the leopard cat (HTLC), (ii) compared it with that of *H. taeniaeformis* from the cat in China (HTCC) and Germany (HTCG), and (iii) tested the hypothesis that three isolates of *H. taeniaeformis* are genetically distinct *via* phylogenetic analyses of amino acid sequence datasets.

## Materials and Methods

### Parasites and DNA Isolation

An adult tapeworm representing *H. taeniaeformis* (designated HTLC) was collected from the small intestine of a leopard cat (*Prionailurus bengalensis*) in Beijing Zoo, China. The tapeworm was washed in physiological saline, identified primarily based on morphological characters using existing keys to species (18), fixed in 70% (v/v) ethanol, and stored at −20°C until use. Total genomic DNA was isolated from this tapeworm using sodium dodecyl sulfate/proteinase K treatment, followed by spin-column purification (Wizard^®^ SV Genomic DNA Purification System, Promega). Furthermore, the specimen was also identified as *H. taeniaeformis* by PCR-based sequencing of 28S rDNA gene and mt *cox*1 gene and had 99.2 and 100% nucleotide homology with those of *H. taeniaeformis* from Norway rat in India and brown rat in China deposited in GenBank (GenBank accession nos. JN020350 and MF380378), respectively.

### Sequencing, Assembling, and Verification

A genomic DNA library (350 bp inserts) was prepared and sequenced by Novogene Bioinformatics Technology Co. Ltd. (Tianjin, China) using the Illumina HiSeq 2500 (250 bp paired-end reads). The clean reads were obtained from raw reads by removing adaptor sequences, highly redundant sequences, and “N”-rich reads, then were assembled into contigs with Geneious v 11.1.5 (19). Preliminary *cox*1 sequences of *H. taeniaeformis* were used as initial references for assembly with the suitable parameters (minimum overlap identity = 99%, minimum overlap = 150 bp, and maximal gap size = 5 bp) (19). The assembly generated a large contig ending with overlapping fragments. As this structure allowed a single circular organization of the mt genome, we assumed that the complete mt genome had been assembled completely. The completeness of the mt genome assembly was further verified by a long PCR experiment using four pairs of primers (Supplementary Figure S1 and Supplementary Table S1), which were designed in the conserved regions. When the additive sizes of amplicons are identical to the length of the assembled contig, it proves that the assembly is correct. The long PCR reaction system consisted of 25 μl: 10.5 μl ddH_2_O, 0.5 μl upstream primer, 0.5 μl downstream primer, 12.5 μl Premix Taq™ (Takara, LA Taq™ V 2.0 plus dye), and 1 μl genomic DNA. Samples were tested in the C1000 Touch™ Thermal Cycler (BioRad, USA) under the following conditions: 94°C for 1 min (initial denaturation), then 98°C for 10 s (denaturation), 54–59°C for 40 s (annealing), and 68°C for 4 min for 35 cycles, with a final extension at 72°C for 8 min.

### Annotation, Visualization, and Sequence Analysis

Protein-coding and rRNA genes were identified by alignment to homologous genes of the previously sequenced mt genome of *H. taeniaeformis* (17) using the MAFFT v7.122 software (20). tRNA genes were identified using ARWEN (21) and tRNAscan-SE (22). MEGA v6.0 (23) was utilized to analyze the amino acid sequences of each protein-coding gene. The map of the mt genome of the *H. taeniaeformis* was figured on the proksee online server (https://proksee.ca/). Pairwise comparisons of the complete mt genomes were made among China isolates (designated HTCC, GenBank accession number: FJ597547), Germany isolates (designated HTCG, GenBank accession number: JQ663994), and China isolates in the present study, such as lengths, identities, A + T content, and codons. The A + T content was computed using DNAStar (v. 5.0). Sequence identities were calculated by ClustalW in Geneious v 11.1.5 (19): cost matrix IUB, gap open cost of 15, and gap extend cost of 6.66.

### Phylogenetic Analyses

A total of 33 mt genome sequences of tapeworms within the family Taeniidae were used for phylogenetic analysis (Table S2), using *Paruterina candelabraria* (GenBank accession number NC039533) as an outgroup (24). Amino acid sequences were single aligned using MAFFT 7.122. The aligned sequences were then concatenated to form a single contig. The poor blocks were excluded from the contig using Gblocks 0.91b (http://phylogeny.lirmm.fr/phylo_cgi/one_task.cgi?task_type=gblocks) using default parameters (25). Phylogenetic analyses were conducted using two methods: Bayesian inference (BI) and maximum likelihood (ML). BI analysis was performed in MrBayes 3.1.1 as described previously (26, 27). ML analysis was performed with PhyML 3.1 as described previously (27, 28). The JTT + I + G + F [lnL = −57,363.05, gamma distribution parameter (*G*) = 0.857, proportion of invariable sites (*I*) = 0.304] model selected by ProtTest 3.4.2 (29) was used based on the Akaike information criterion (AIC). Phylograms were drawn using the program FigTree v.1.4.

### Analysis of *Cox*1 Gene Sequence

The newly generated sequences in the present study and previously published *cox*1 sequences of *H. taeniaeformis* (*n* = 84) from different hosts and regions (13), were aligned using the software MAFFT 7.122. The nucleotide sequence of these sequences was included in the present analysis, using *H. krepkogorski* (GenBank accession number NC021142) as an outgroup (6). The phylogenetic tree was constructed with Mega v 6.0 (23) using the Neighbor–Joining (NJ) method. The Kimura 2-parameter (K2P) model was the most suitable model, with 1,000 bootstrap replicates.

## Results

Original data of this study including nucleotide sequence alignments (*cox*1 complete haplotypes dataset) are available at Mendeley Data (http://dx.doi.org/10.17632/b3knx84j6n.1).

### Genome Content and Organization

**Figure 1:**
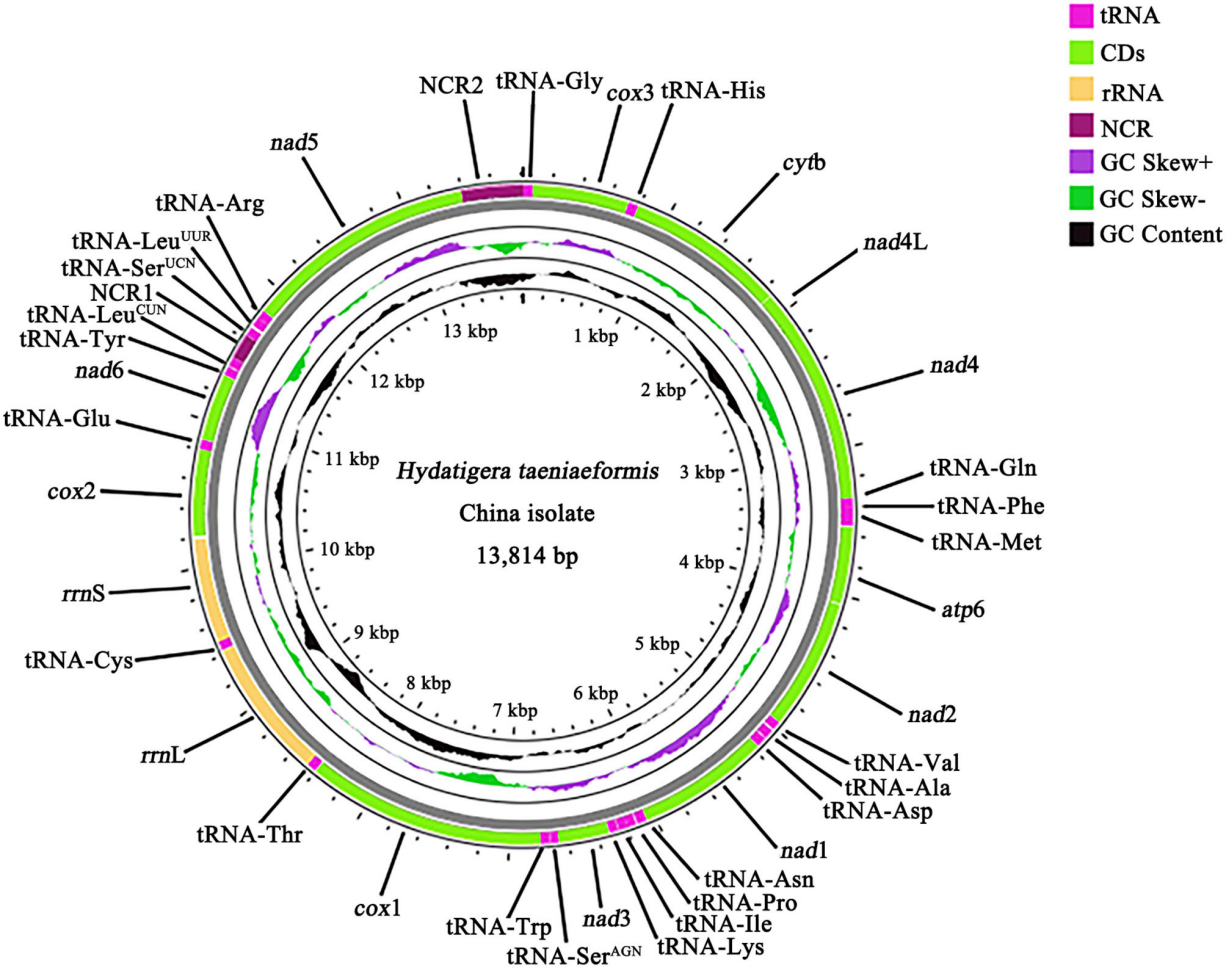
The organization of mitochondrial genome of *Hydatigera taeniaeformis* China isolate. Gene scaling is only approximate. “NCR1” refers to a short non-coding region and “NCR2” refers to a long non-coding region.

We sequenced the *H. taeniaeformis* genome and produced over 3 Gb of Illumina short-read sequence datasets. A total of 20,634,954 × 2 raw reads were generated and 19,826,948 × 2 clean reads were obtained for assembly of the mt genome. The complete mt genome sequence of HTLC (GenBank accession no. ON055368) was 13,814 bp in size (Figure 1) and have a high A + T content (73.2%). It contains 12 protein-coding genes (*cox*1–3, *nad*1–6, *nad*4L, *atp*6, and *cyt*b), 22 tRNA genes, two rRNA genes (*rrn*S and *rrn*L), and two non-coding (control or AT-rich) regions (NCR), but lacks an *atp*8 gene (Figure 1). The mt genome arrangement of this tapeworm is the same as that of *Spirometra erinaceieuropaei* and *Raillietina tetragona* (27, 30).

### Annotation

All of the 12 protein-coding genes begin with ATG (*cyt*b, *atp*6, *nad*4L, *nad*1–3, *cox*1, *cox*2, and *nad*5), ATT (*cox*3), or GTG (*nad*4 and *nad*6) as their start codons. Six of the 12 genes appear to use TAA (*nad*1, *nad*2, *nad*5, *nad*6, *cox*3, and *cyt*b) or TAG (*atp*6, *nad*4, and *nad*4L) as the stop codons. Three genes (*cox*1, *cox*2, and *nad*3) end with incomplete stop codon T (Table 1). The 22 tRNA genes varied from 59 to 68 bp in size (Table 1). Secondary structures predicted for the 22 tRNA genes of HTLC (not shown) are similar to those of other tapeworms *T. solium and T. asiatica* (31). The *rrn*L gene of this mt genome is located between tRNA-Thr and tRNA-Cys, and *rrn*S gene is located between tRNA-Cys and *cox*2. The length of *rrn*L and *rrn*S genes is 953 and 724 bp, respectively. The A + T content of *rrn*L and *rrn*S is 72.8 and 71.9%, respectively. Two NCRs in the mt genome were inferred. The NCR1 (172 bp) is located between the tRNA-Leu^CUN^ (L1) and tRNA-Ser^UCN^ (S2), and has an A+T content of 79.7%. The NCR2 (418 bp) is located between the *nad*5 and tRNA-Gly and has an A+T content of 79.5%.

**Table 1:** The organization of the mitochondrial (mt) genome of *Hydatigera taeniaeformis*.

**Gene/region**	**Positions**	**Size (bp)**	**Number of aa^a^**	**Ini/Ter codons^b^**	**Anticodons**	**In^c^**
tRNA-Gly (G)	1–65	65			TCC	0
*cox*3	66–713	648	216	ATT/TAA		+3
tRNA-His (H)	717–784	68			GTG	+2
*cyt*b	787–1,860	1,074	358	ATG/TAA		+4
*nad*4L	1,865–2,125	261	87	ATG/TAG		−34
*nad*4	2,092–3,342	1,251	417	GTG/TAG		+0
tRNA-Gln (Q)	3,343–3,406	64			TTG	−3
tRNA-Phe (F)	3,404–3,467	64			GAA	−5
tRNA-Met (M)	3,463–3,528	66			CAT	+9
*atp*6	3,538–4,056	519	173	ATG/TAG		+6
*nad*2	4,063–4,950	888	296	ATG/TAA		+1
tRNA-Val (V)	4,952–5,012	61			TAC	+11
tRNA-Ala (A)	5,024–5,089	66			TGC	+5
tRNA-Asp (D)	5,095–5,160	66			GTC	+6
*nad*1	5,167–6,060	894	298	ATG/TAA		0
tRNA-Asn (N)	6,061–6,128	68			GTT	+7
tRNA-Pro (P)	6,136–6,196	61			TGG	−1
tRNA-Ile (I)	6,196–6,259	64			GAT	+2
tRNA-Lys (K)	6,262–6,322	61			CTT	+4
*nad*3	6,327–6,666	340	113	ATG/T		0
tRNA-Ser^AGN^ (S1)	6,667–6,725	59			GCT	+1
tRNA-Trp (W)	6,727–6,788	62			TCA	0
*cox*1	6,789–8,400	1,612	537	ATG/T		+8
tRNA-Thr (T)	8,409–8,470	62			TGT	0
*rrn*L	8,471–9,423	953				+8
tRNA-Cys (C)	9,432–9,491	60			GCA	−21
*rrn*S	9,471–10,194	724				+23
*cox*2	10,218–10,800	583	194	ATG/T		+0
tRNA-Glu (E)	10,801–10,867	67			TTC	+3
*nad*6	10,871–11,320	450	150	GTG/TAA		+3
tRNA-Tyr (Y)	11,324–11,385	62			GTA	+6
tRNA-Leu^CUN^ (L1)	11,392–11,454	63			TAG	0
Non-coding region (NCR1)	11,455–11,626	172				0
tRNA-Ser^UCN^ (S2)	11,627–11,688	62			TCA	+17
tRNA-Leu^UUR^ (L2)	11,706–11,767	62			TAA	−1
tRNA-Arg (R)	11,767–11,825	59			ACG	+2
*nad*5	11,828–13,396	1,569	523	ATG/TAA		0
Non-coding region (NCR2)	13,397–13,814	418				0

a *The inferred length of amino acid (aa) sequence of 12 protein-coding genes*.

b *Ini/Ter codons, initiation and termination codons*;

c *In, intergenic nucleotides*.

### Comparative Mt Genomic Analyses of HTLC With HTCC and HTCG

The mt genome sequence of HTLC was 13,814 bp in length, 167 bp longer than that of HTCC, and 74 bp longer than that of HTCG. The arrangement of the mt genes (i.e., 12 protein-coding genes, 22 tRNA genes, and two rRNA genes) and two NCRs was the same. Across the entire mt genome (except for the NCR), the sequence difference was 3.3% between HTLC and HTCC, 12.0% between HTLC and HTCG, and 12.1% between HTCC and HTCG. The greatest nucleotide variation among the three isolates of *H. taeniaeformis* was in the *nad*3 gene, whereas the least difference was detected in the *cox*3 (Table 2). Amino acid sequences inferred from individual mt protein genes of HTLC were compared with those of HTCC and HTCG. The difference across amino acid sequences of the 12 protein genes between the HTLC and HTCC was 2.3%, 10.0% between HTLC and HTCG, and 10.6% between HTCC and HTCG. The amino acid sequence differences among three isolates of *H. taeniaeformis* ranged from 0.4 to 18.3%, with COX2 being the most conserved and NAD3 the least conserved protein. For the 22 tRNAs, the sequence difference was 3.6% between HTLC and HTCC, was 5.7% between HTLC and HTCG, and was 7.5% between HTCC and HTCG. For the *rrn*L and *rrn*S genes, the sequence difference was 3.1 and 6.1% between HTLC and HTCC, 11.5 and 13.9% between HTLC and HTCG, and 10.0 and 9.4% between HTCC and HTCG, respectively (Table 2).

**Table 2:** Nucleotide (nt) and/or predicted amino acid (aa) sequence differences in mt genes *H. taeniaeformis* from the leopard cat (HTLC) (A) and *H. taeniaeformis* from the cat in China (HTCC) (B); HTLC (A) and *H. taeniaeformis* from the cat in Germany (HTCG) (C); HTCC (B) and HTCG (C) upon pairwise comparison.

**Gene/region**	**Nt sequence length**			**Nt difference (%)**			**Number of aa**			**aa difference (%)**		
	**A**	**B**	**C**	**A/B**	**A/C**	**B/C**	**A**	**B**	**C**	**A/B**	**A/C**	**B/C**
*atp*6	519	519	519	3.9	12.7	13.1	172	172	172	2.3	11.1	11.1
*cox*1	1,612	1,612	1,635	3.1	10.7	10.8	537	537	544	0.4	2.2	2.8
*cox*2	583	582	583	3.8	6.4	7.0	194	193	194	1.0	1.6	1.56
*cox*3	648	645	645	2.9	11.0	11.2	216	214	214	1.4	6.9	6.5
*cyt*b	1,074	1,047	1,068	5.9	14.1	16.7	358	348	355	5.0	10.9	13.5
*nad*1	894	894	894	3.5	12.5	13.3	298	297	297	2.7	12.1	12.5
*nad*2	888	891	888	4.6	14.5	14.3	296	296	295	3.0	12.1	10.8
*nad*3	340	340	348	5.6	17.8	17.8	113	113	115	7.1	17.4	18.3
*nad*4	1,251	1,251	1,251	4.2	15.0	15.4	416	416	416	2.6	14.7	14.2
*nad*4L	261	261	261	3.8	14.6	14.9	86	86	86	2.3	11.6	11.6
*nad*5	1,569	1,569	1,569	4.3	15.9	15.6	523	522	522	3.8	14.5	14.2
*nad*6	450	448	453	4.2	15.5	16.3	150	149	150	4.7	12.6	16.0
*rrn*S	724	717	725	6.1	13.9	9.4	–	–	–	–	–	–
*rrn*L	953	959	959	3.1	11.5	10.0	–	–	–	–	–	–
All 22tRNA	1,392	1,410	1,399	3.6	5.7	7.5	–	–	–	–	–	–

### Phylogenetic Analyses

**Figure 2:**
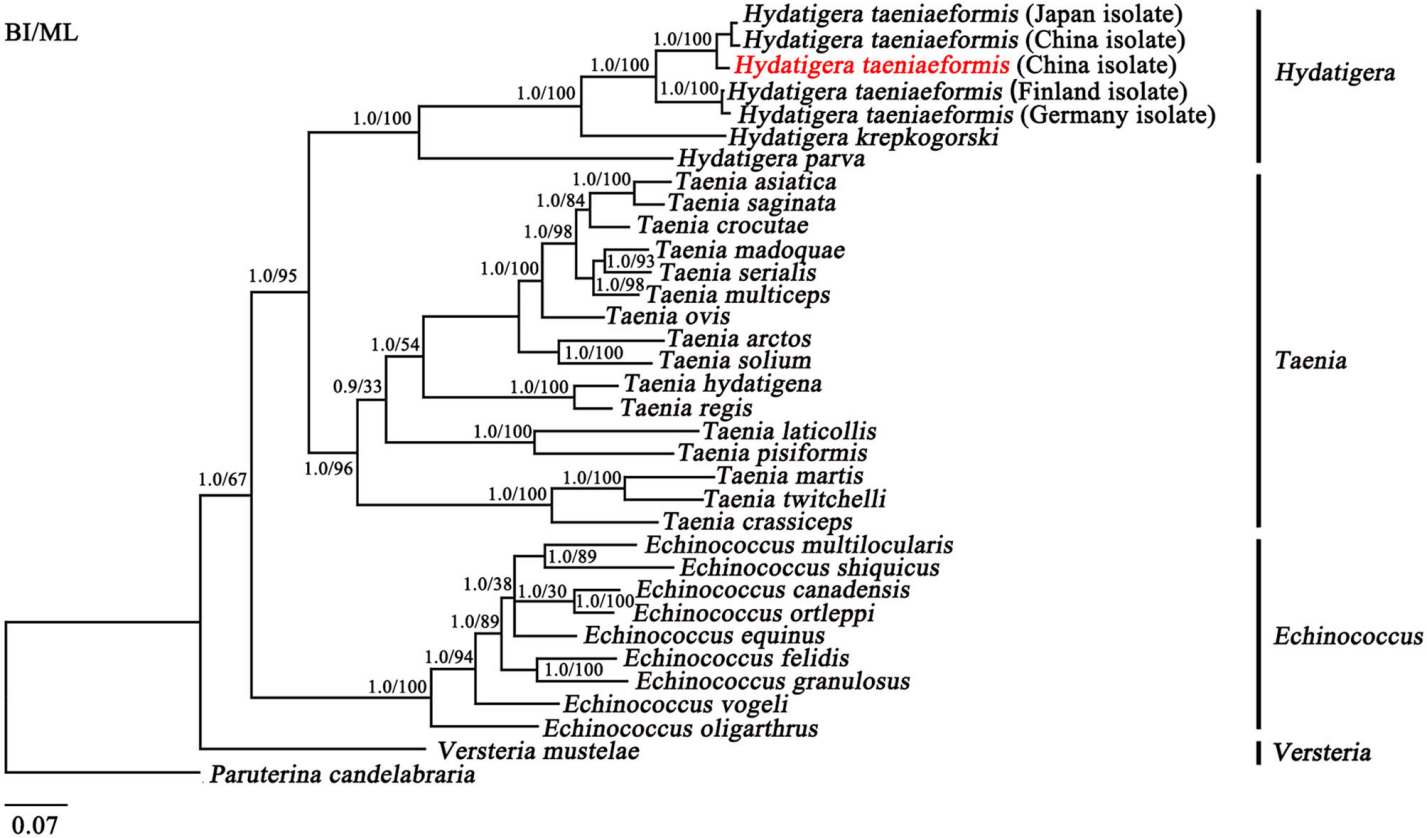
Inferred phylogenetic relationship among species from the family Taeniidae. The concatenated amino acid sequences of 12 mitochondrial protein-coding genes were analyzed utilizing Maximum likelihood (ML) and Bayesian analysis (BI), using Paruterina candelabraria as an outgroup. Hydatigera taeniaeformis in the red font is China isolate from the leopard cat in the present study.

The topologies of the trees using BI and ML were identical (Figure 2). In the tree, four major clades were recovered within the family Taeniidae: [(*Hydatigera* + *Taenia*) + *Echinococcus*] and *Versteria* from the monophyletic groups. Within the *Hydatigera*, all *Hydatigera* isolates clustered together with high statistical support (Bpp = 1; Bf = 100), supporting that the genus *Hydatigera* is valid (32). In the present study, the separation of *H. taeniaeformis* isolates from different hosts and countries was shown to be into two distinct clades with strong support (Bpp = 1; Bf = 100; Figure 2). The *H. taeniaeformis* isolates from cats in Japan, and China and leopard cat in China were clustered in the same clade, while *H. taeniaeformis* isolates from cats in Finland and Germany were clustered in the other clade, which was sister taxa with the former (Figure 2). The differences between the two distinct clades are slightly longer (looking at branch lengths) than between *T. asiatica* and *T. saginata* (6) and similar to *Echinococcus* spp. (33–35) (Figure 2). In addition, phylogenetic analyses based on the mt *cox*1 sequences among *H. taeniaeformis* (*n* = 84) from different hosts and geographical regions revealed three distinct clades (Supplementary Figure S2), which are consistent with a previous study (13).

## Discussion

The broad consensus on the presence of cryptic species within cat tapeworm *H. taeniaeformis* (6, 13, 17, 36), but their taxonomic status remains unknown. As described previously, in the morphological features, the variations were embodied in the number of proglottids, the mean number of rostellar hooks, the small hooks of adult worms and metacestodes, the direction of the small hooks, the length of the cirrus sac, and the mean number and location of testes (10). Moreover, in terms of molecular evidence, a previous study reported that the level of nucleotide variation in the *cox*1 gene within the genus *Hydatigera* (formerly referred to as *Taenia*) is about 6.3–15.6% (37). Another study showed the *cox*1 gene sequence of a new cryptic species *H. taeniaeformis* was distinct from those of other isolates (9.0–9.5%) (12). In the present study, the sequence variations (12.0 and 12.1%) detected in the whole mt genome were consistent with previous results mentioned above in part of the mt *cox*1 gene among *H. taeniaeformis* from different regions and hosts (12, 13). These findings indicate that in terms of classification, our results support our proposal that the *H. taeniaeformis* species complex consists of at least two cryptic species. In addition, prior studies have indicated that mtDNA sequence variation between closely-related nematode species was typically in the range of 10–20% (12, 38).

Phylogenetic analyses of *H. taeniaeformis* provided additional evidence that *H. taeniaeformis* represents closely but distinct taxa. Taken together, the molecular evidence presented here supports the hypothesis that *H. taeniaeformis* represents a species complex (13, 17). The taxonomy of *H. taeniaeformis* is often insufficient based on morphological features and their hosts or geographical origins; therefore, accurate identification and differentiation of *H. taeniaeformis* can sometimes be challenging (7, 13, 36). In the present study, the characterization of the mt genome of HTLC in China also stimulates a reassessment of the phylogenetic relationships of *H. taeniaeformis* using mt genomic/proteomic datasets. Mt genomic sequences are useful molecular markers to study the species identification, genetic structure, and phylogenetic analyses of many worms, especially for searching potential cryptic species (17, 38). Therefore, in the present study, the analyses of mt genomic sequences provided insight into the phylogenetic relationships of *H. taeniaeformis*; however, some *H. taeniaeformis* isolates from cats in Italy (clade C) (36, 39) are either not represented or underrepresented (13). Therefore, further studies are required to resolve the taxonomic classification of *H. taeniaeformis* species complex by sequencing the mt genomic sequences of additional *H. taeniaeformis* from different countries and hosts.

## Conclusions

The present study sequenced the complete mt genome sequence of the cat tapeworm HTLC in China. The comparative mt genomic analysis provided robust genetic evidence that *H. taeniaeformis* represents a species complex. The novel mt genomic datasets provide useful markers for further studies of the taxonomy and systematics of cat tapeworm *H. taeniaeformis*.

## Data Availability Statement

The datasets presented in this study can be found in online repositories. The names of the repository/repositories and accession number(s) can be found at: https://www.ncbi.nlm.nih.gov/genbank/, ON055368.

## Ethics Statement

This study was approved by the Animal Ethics Committee of Hunan Agricultural University (No. 43321503).

## Author Contributions

Y-TF, G-HL, and H-MW: conceptualization, design, revision critically, and analysis. H-MW: experiment and drafted the manuscript. RL and Y-PD: study implementation, and manuscript preparation. All authors read and approved the final manuscript.

## Funding

This study was supported in part, by the Training Program for Excellent Young Innovators of Changsha (Grant No. KQ2106044).

## Conflict of Interest

The authors declare that the research was conducted in the absence of any commercial or financial relationships that could be construed as a potential conflict of interest.

## Publisher's Note

All claims expressed in this article are solely those of the authors and do not necessarily represent those of their affiliated organizations, or those of the publisher, the editors and the reviewers. Any product that may be evaluated in this article, or claim that may be made by its manufacturer, is not guaranteed or endorsed by the publisher.

## Supplementary Material

Supplementary Figure S1 PCR verification of the mt genome of *Hydatigera taeniaeformis* China isolate. Lane M: DL5000 DNA marker. Lane 1–4: cox3-nad1, nad1-cox1, nad6-cox3, and nad6-cox3.

Supplementary Figure S2 Inferred phylogenetic relationship based on *cox1* sequences of *H. taeniaeformis* (*n* = 84) from different hosts and regions. Clade A consisted of 39 haplotypes (designated as A1–A39, A37 in the red font is the *cox1* fragment of *H. taeniaeformis* in this study), clade B included 23 haplotypes (B1–B23), and clade C contained 22 haplotypes (C1–C22), using Neighbor-Joining (NJ) method, with an outgroup *H. krepkogorski*.

Supplementary Dataset Mitochondrial DNA data. Nucleotide sequence alignments (*cox1* complete haplotypes dataset). (http://dx.doi.org/10.17632/b3knx84j6n.1).
