# Supplementary figures and images for: Comparative Mitochondrial Genomic Analysis Robustly Supported That Cat Tapeworm Hydatigera taeniaeformis (Platyhelminthes: Cestoda) Represents a Species Complex
Source: Front Vet Sci. 2022 Jun 22;9:931137. doi: 10.3389/fvets.2022.931137 (PMC9258744; doi:10.3389/fvets.2022.931137)

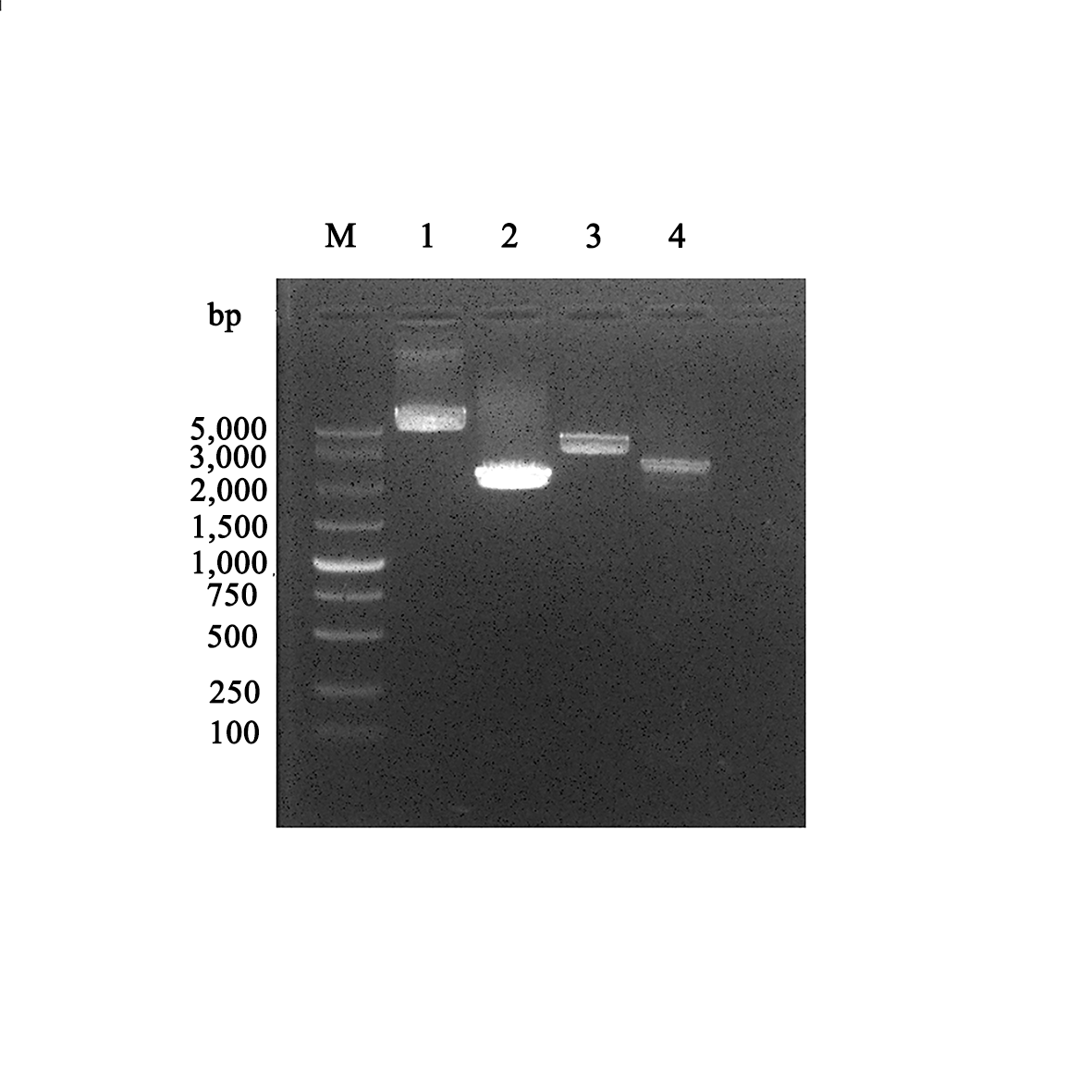

Supplement: Supplementary Figure S1 — PCR verification of the mt genome of Hydatigera taeniaeformis China isolate. Lane M: DL5000 DNA marker. Lane 1–4: cox3-nad1, nad1-cox1, nad6-cox3, and nad6-cox3. [file Image_1.TIF]

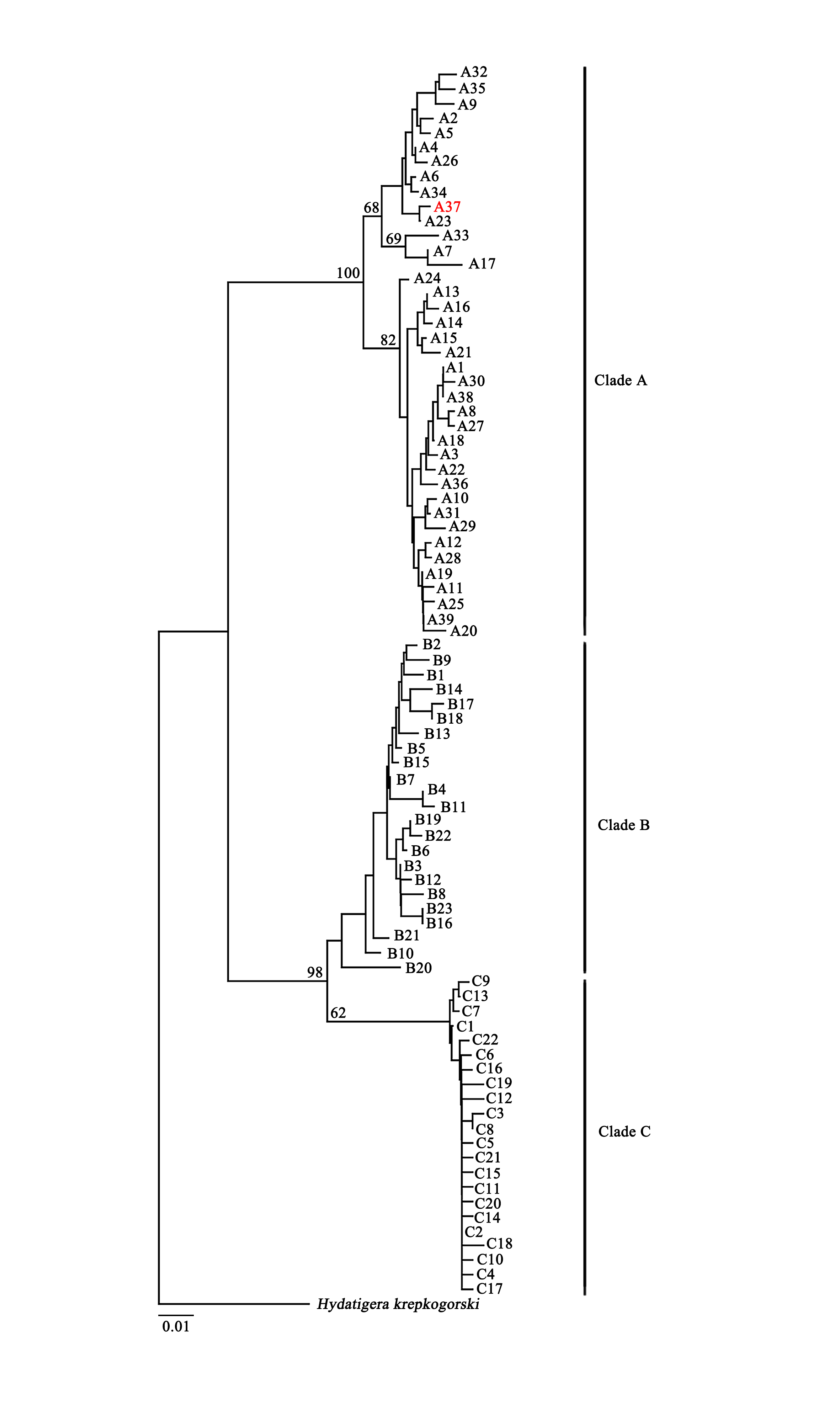

Supplement: Supplementary Figure S2 — Inferred phylogenetic relationship based on cox1 sequences of H. taeniaeformis (n = 84) from different hosts and regions. Clade A consisted of 39 haplotypes (designated as A1–A39, A37 in the red font is the cox1 fragment of H. taeniaeformis in this study), clade B included 23 haplotypes (B1–B23), and clade C contained 22 haplotypes (C1–C22), using Neighbor-Joining (NJ) method, with an outgroup H. krepkogorski. [file Image_2.TIF]
