# Supplementary material for: Comparative Mitochondrial Genomic Analysis Robustly Supported That Cat Tapeworm Hydatigera taeniaeformis (Platyhelminthes: Cestoda) Represents a Species Complex
Source: Front Vet Sci. 2022 Jun 22;9:931137. doi: 10.3389/fvets.2022.931137 (PMC9258744; doi:10.3389/fvets.2022.931137)
Supplement: Supplementary file 3 [file Data_Sheet_1.docx]

**TABLE S1 |** PCR primers used to verify the mitochondrial genome of *Hydatigera taeniaeformis*.

| **Primer no.** | **Sequence (5’ to 3’)** | **Gene/Region** | **Size (bp)** |
| --- | --- | --- | --- |
| 1 | F:GGTTGTTTGTATTTAGCGAAGTTATGG | *cox*3-*nad*1 | 5,196 |
|  | R:CAAAGCATTGAAAACTCACCTATCT |  |  |
| 2 | F:GTAAGGGTCCTAATAAGGTTGGT | *nad*1-*cox*1 | 2,243 |
|  | R:ACCCAGGAAGAATCAATACATAC |  |  |
| 3 | F:TAGTTTGTTTAGGAAGAAGGGTG | *cox*1-*nad*6 | 3,687 |
|  | R:AAGAGTTTACAACCCTCCACATT |  |  |
| 4 | F:GATGTTGATGTTTGGGTTTGTTG | *nad*6-*cox*3 | 2,902 |
|  | R:GTAACCGTTATACTAGAACCAAG |  |  |

**TABLE S2 |** Mitochondrial genome sequences of the family **Taeniidae** sequenced completely prior to the present study and used for phylogenetic analysis.

| **Family** | **Species** | **Size (bp)** | **GenBank accession number** |
| --- | --- | --- | --- |
| **Taeniidae** | ***Echinococcus canadensis*** | 13,731 | MT166289 |
|  | ***Echinococcus equinus*** | 13,598 | AF346403 |
|  | ***Echinococcus felidis*** | 13,632 | NC021144 |
|  | ***Echinococcus granulosus*** | 13,605 | KJ559023 |
|  | ***Echinococcus multilocularis*** | 13,738 | NC000928 |
|  | ***Echinococcus oligarthrus*** | 13,791 | NC009461 |
|  | ***Echinococcus ortleppi*** | 13,717 | NC011122 |
|  | ***Echinococcus shiquicus*** | 13,807 | NC009460 |
|  | ***Echinococcus vogeli*** | 13,750 | NC009462 |
|  | ***Hydatigera taeniaeformis*** | 13,853 | NC037071 |
|  | ***Hydatigera krepkogorski*** | 13,792 | NC021142 |
|  | ***Hydatigera parva*** | 13,482 | NC021141 |
|  | ***Hydatigera taeniaeformis*** | 13,822 | AP017671 |
|  | ***Hydatigera taeniaeformis*** | 13,647 | FJ597547 |
|  | ***Hydatigera* *taeniaeformis*** | 13,740 | JQ663994 |
|  | ***Hydatigera* *taeniaeformis*** | 13,814 | ON055368 |
|  | ***Taenia arctos*** | 13,650 | NC024590 |
|  | ***Taenia asiatica*** | 13,703 | AF445798 |
|  | ***Taenia crassiceps*** | 13,503 | AF216699 |
|  | ***Taenia crocutae*** | 13,711 | AB905201 |
|  | ***Taenia hydatigena*** | 13,494 | MT784896 |
|  | ***Taenia laticollis*** | 13,483 | NC021140 |
|  | ***Taenia madoquae*** | 13,689 | NC021139 |
|  | ***Taenia martis*** | 13,536 | NC020153 |
|  | ***Taenia multiceps*** | 13,693 | GQ228818 |
|  | ***Taenia ovis*** | 13,704 | NC021138 |
|  | ***Taenia pisiformis*** | 13,387 | GU569096 |
|  | ***Taenia regis*** | 13,489 | AB905198 |
|  | ***Taenia saginata*** | 13,670 | AY684274 |
|  | ***Taenia serialis*** | 13,688 | NC021457 |
|  | ***Taenia solium*** | 13,709 | NC004022 |
|  | ***Taenia twitchelli*** | 13,519 | NC021093 |
|  | ***Versteria mustelae*** | 13,582 | AB732957 |
